# Chromatin Proteomics to Study Epigenetics — Challenges and Opportunities

**DOI:** 10.1074/mcp.R120.002208

**Published:** 2021-02-05

**Authors:** Guido van Mierlo, Michiel Vermeulen

**Affiliations:** Department of Molecular Biology, Faculty of Science, Radboud Institute for Molecular Life Sciences, Oncode Institute, Radboud University Nijmegen, Nijmegen, the Netherlands

**Keywords:** chromatin, immunoprecipitation, MS, proteomics, transcription factors, AP, affinity purification, ChEP, Chromatin Enrichment for Proteomics, ChIP–SICAP, the ChIP and selective isolation of chromatin-associated proteins, ChroP, chromatin proteomics, DEMAC, density-based enrichment for MS analysis of chromatin, DIA, data-independent acquisition, HRP, horseradish peroxidase, MNase, micrococcal nuclease, NuRD, nucleosome remodeling and deacetylase, PPIs, protein–protein interactions, RIME, Rapid Immunoprecipitation MS of Endogenous proteins, TFs, transcription factors, XL–MS, crosslinking–MS

## Abstract

Regulation of gene expression is essential for the functioning of all eukaryotic organisms. Understanding gene expression regulation requires determining which proteins interact with regulatory elements in chromatin. MS-based analysis of chromatin has emerged as a powerful tool to identify proteins associated with gene regulation, as it allows studying protein function and protein complex formation in their *in vivo* chromatin-bound context. Total chromatin isolated from cells can be directly analyzed using MS or further fractionated into transcriptionally active and inactive chromatin prior to MS-based analysis. Newly formed chromatin that is assembled during DNA replication can also be specifically isolated and analyzed. Furthermore, capturing specific chromatin domains facilitates the identification of previously unknown transcription factors interacting with these domains. Finally, in recent years, advances have been made toward identifying proteins that interact with a single genomic locus of interest. In this review, we highlight the power of chromatin proteomics approaches and how these provide complementary alternatives compared with conventional affinity purification methods. Furthermore, we discuss the biochemical challenges that should be addressed to consolidate and expand the role of chromatin proteomics as a key technology in the context of gene expression regulation and epigenetics research in health and disease.

## Highlights

• An overview of proteomics methods to study chromatin and gene regulation.
• Strength and limitations of the different approaches are highlighted.
• An outlook on the outstanding challenges for chromatin proteomics.
• Future directions for chromatin proteomics are discussed.

## Graphical abstract

**Figure:**
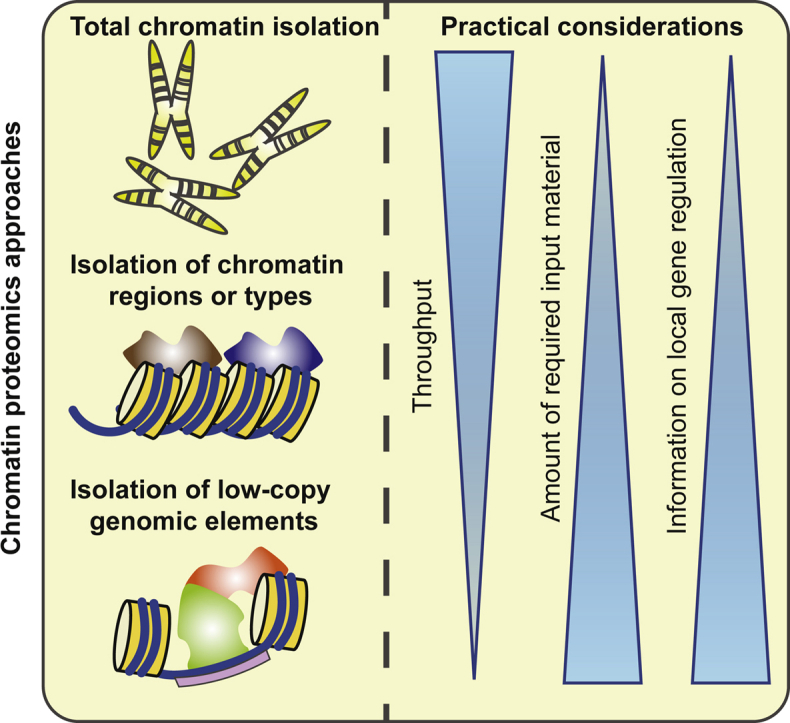

## In Brief

MS-based analysis of chromatin has emerged as a powerful tool to identify proteins associated with gene regulation. Total chromatin isolated from cells can be directly analyzed using MS, further fractionated into transcriptionally active and inactive chromatin, enriched for specific compartment or regions, and potentially used for single-locus isolation. This review highlights recent advances and discusses current challenges that should be addressed to further advance the field of chromatin proteomics.

Chromatin, which is present in every eukaryotic cell, is a complex assembly of DNA and proteins, which plays a central role in the regulation of gene expression. Gene expression regulation involves recruitment of proteins (so-called transcription factors [TFs]) to regulatory regions in the genome, such as promoters and enhancers. Consequently, these TFs recruit a multitude of factors including chromatin modifying and chromatin remodeling complexes that, depending on their function, mediate either transcriptional activation or silencing of a gene. To comprehend how gene expression is regulated, chromatin research aims to identify all TF-binding sites, how TFs and regulatory protein complexes are recruited to those sites, and how this affects the local chromatin environment. To determine genome-wide binding for chromatin-associated proteins of interest, CHromatin Immuno Precipitation followed by sequencing and cleavage under targets and release using nuclease are currently the most widely used methods (1, 2). These techniques use antibodies against a protein of interest, followed by immunoprecipitation (IP) and sequencing of the copurifying DNA fragments, allowing pinpointing the location of these proteins on a genome-wide scale. Such assays are frequently complemented with techniques such as Assay for Transposase-Accessible Chromatin using sequencing to identify accessible chromatin regions (3) and RNA-Seq to determine the expression state of these genes. Together, these assays allow determining the genome-wide binding of proteins and whether these binding sites are associated with active or inactive chromatin. In addition, novel regulatory proteins binding those regions can be computationally predicted based on the underlying DNA sequence (4, 5, 6).

Genome-wide profiling technologies are crucial for our understanding of gene regulation. However, these techniques do not provide an unbiased and comprehensive view of chromatin since they are limited to studying one or a few proteins at a time and are thus limited with respect to their ability to identify novel chromatin regulatory proteins. In addition, genomics techniques (1) are slightly biased toward active chromatin regions (7); (2) computational predictions for TF binding are dependent on pre-existing knowledge regarding DNA-motif–based predictions; (3) provide limited knowledge regarding interaction motifs for proteins that do not interact with DNA directly, such as binders of nucleosomes and histone modifications; and (4) are biased toward proteins with an expected chromatin function, thus neglecting potential “moonlighting proteins.” To overcome these limitations, high-resolution MS approaches have been developed to study protein function and protein–protein interactions (PPIs) in a more comprehensive and unbiased manner (8). Adaptation of such workflows to study epigenetic processes, and development of innovative protocols, has significantly enhanced the toolbox available to study chromatin-based processes in health and disease. These recent advances will be the focus of this review, and we highlight the advantage and limitations of these workflows. We further discuss the outstanding challenges and opportunities and how these could be addressed. While this review mainly focuses on TFs, many of the discussed techniques can also be adapted to study other chromatin features such as histone modifications.

## Chromatin Enrichment Strategies for MS

**Fig. 1:**
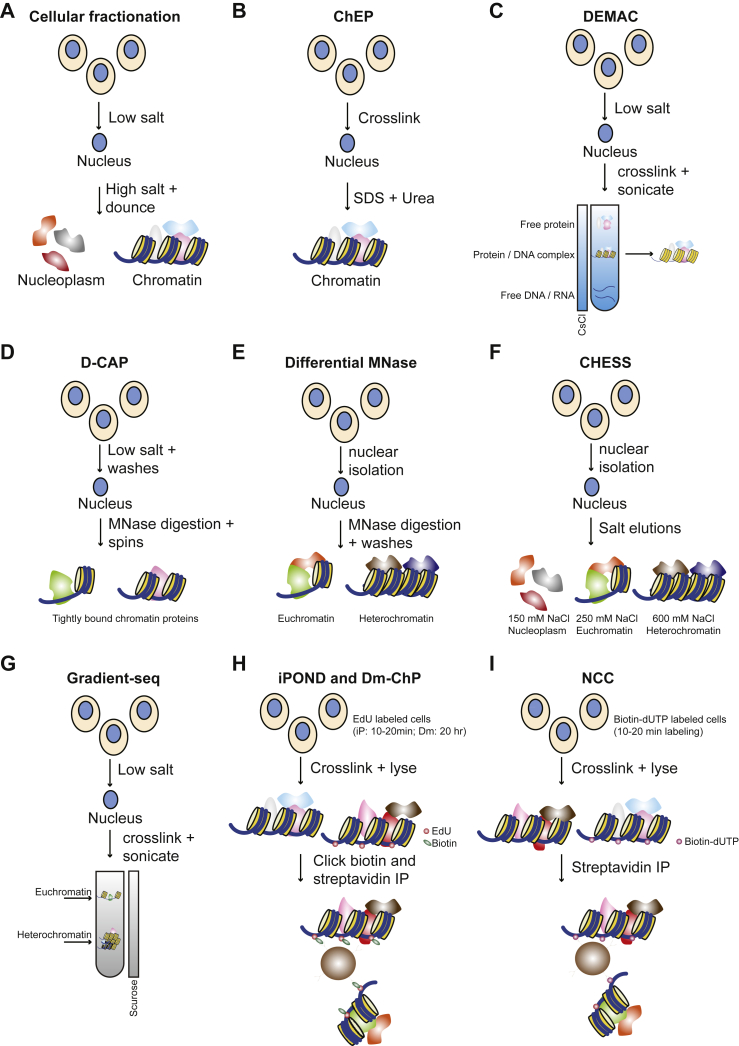
**Approaches to isolate chromatin for MS**. *A*, crude fractionation of native cells in cytoplasm (obtained after the low salt incubation), nucleus, and chromatin. *B*, crosslinking followed by SDS–urea-mediated chromatin isolation. *C*, chromatin separation from crosslinked nuclear extract using CsCl ultracentrifugation. In a CsCl gradient, free DNA ends up as pellet, free protein in the top of the gradient, and protein–DNA complexes in the middle. This middle fraction can be isolated by inserting a needle in the side of the tube. *D*, isolation of (mainly) euchromatic proteins using MNase. *E*, separation of euchromatin and heterochromatin using brief MNase digestion and washes. Note that D-CAP first washes nuclei with salts to remove nontightly bound proteins, and after MNase digestion, just proteins “tightly” bound to chromatin should be released. Conversely, in the differential MNase digestion, a brief digestion results in release of mononucleosome fragments (euchromatic regions) and a second wash rather elutes more heterochromatic proteins. *F*, separation of nucleoplasmic, euchromatic, and heterochromatic proteins using different NaCl concentrations. *G*, chromatin isolation using sucrose density ultracentrifugation. In this gradient, the denser, or heavier, proteins and complexes end up lower in the gradient. Taking fractions using a needle at several positions thus isolates chromatin sections from different sizes. Note that this method has only been used to do proteomics on the heterochromatin fraction. *H* and *I*, incorporation of thymidine analogs to purify nascent (iPOND and NCC) or all (Dm-ChP) chromatin. CsCl, cesium chloride; D-CAP, differential chromatin-associated proteins; Dm-ChP, DNA-mediated chromatin pull-down; iPOND, isolation of Proteins On Nascent DNA; MNase, micrococcal nuclease; NCC, nascent chromatin capture.

Holistic approaches provide a powerful starting point in studies aimed at comparing two or more conditions against each other, such as healthy *versus* diseased cells. Purifying chromatin followed by MS-based profiling allows identification of the chromatin-bound proteome in a cell-type or disease-specific manner. The advantage of this approach compared with MS-based analysis of whole cell proteomes, or even nuclear proteomes, is that it allows measuring chromatin-associated factors that are typically low abundant and difficult to identify without extensive sample fractionation prior to LC–MS (9). Initial efforts to identify the chromatin-associated proteome used crude cellular fractionation to obtain a native chromatin fraction (Fig. 1*A*), which proved highly informative in defining novel TFs associated with overexpression of the oncogene *c-Myc* (10). Subcellular fractionation for chromatin isolation has an added benefit, namely that the cytoplasmic and the nuclear fraction can be analyzed separately using proteomics, allowing investigation of, for example, protein translocation between the cytoplasm and the chromatin (11). This is an important tool to discover proteins that might be normally sequestered in the cytoplasm but upon cellular or environmental changes translocate to the nucleus to induce gene expression. A notable example includes the Yes-associated protein and transcriptional coactivator with PDZ-binding motif proteins, which are the effector modules of the hippo signaling pathway. Their nucleocytoplasmic distribution is a key determinant to their activity, and aberrant nuclear localization of Yes-associated protein/transcriptional coactivator with PDZ-binding motif has been observed in numerous cancers (12).

While native chromatin purifications have been adopted to obtain new insights into various biological processes (10, 11, 13), these crude extraction approaches are prone to contamination by cytoplasmic proteins (9). The Chromatin Enrichment for Proteomics (ChEP) method, which relies on formaldehyde crosslinking prior to biochemical extraction of the chromatin, is a relatively simple procedure to reduce the contamination of cytoplasmic proteins (14) (Fig. 1*B*). As such, ChEP has been successfully used to study the chromatin of a multitude of cell types of different organisms (*e.g.*, (15, 16, 17, 18)). However, the chromatin remains a biochemically challenging organelle, likely owing to its highly charged nature (18), and the ChEP procedure still results in cytosolic contamination such as mitochondrial proteins (19). A more recent adaptation of crosslinked chromatin enrichment comprises density-based enrichment for MS analysis of chromatin (DEMAC), which relies on crosslinking nuclei, lysis, and subsequent ultracentrifugation of the crosslinked lysate in a buoyant density gradient made from cesium chloride (20) (Fig. 1*C*). The use of nuclear isolation prior to crosslinking and maintaining chromatin in soluble form together may result in lower contamination from cytoplasmic proteins crosslinked to chromatin. Thus, while technically more challenging than ChEP because of the requirement for ultracentrifugation, DEMAC has the potential to enable the characterization of the chromatin-associated proteome with low levels of contaminants. DEMAC is therefore a valuable addition to the toolbox available for studying the chromatin-associated proteome in different cellular contexts.

## Separation of Euchromatin and Heterochromatin for Proteomics

In addition to protocols that are used to isolate the complete chromatin fraction from cells, several fractionation approaches have been developed to separate open or “transcriptionally active” regions, referred to as euchromatin, from closed or “transcriptionally inactive” regions, referred to as heterochromatin. While ~30% of the human genome consists of regulatory elements (21), only a small subset of these are “active” in a given cell type. In MS-based analyses of crude chromatin fractions, abundant structural chromatin proteins therefore mask low-abundant regulatory proteins that interact with these active sites. Selective enrichment of euchromatin or heterochromatin regions of the genome thus serves a dual purpose: on the one hand, it classifies chromatin proteins as being associated with active or repressive chromatin, whereas on the other hand facilitating the detection of low-abundant TFs that only bind a (sub)set of euchromatic regions. Such a separation can be achieved with both native and crosslinked chromatin preparations. Native chromatin can be exposed to detergents to exploit the strength of protein binding to chromatin, which depends on chromatin features, including binding of cofactors and structural variation (22). Digestion of native nuclei with micrococcal nuclease (MNase) using different amounts or time points will first release proteins associated with euchromatin and subsequently those associated with condensed heterochromatin, owing to the preference of MNase for nucleosome-free regions (23, 24). Coupled to proteomics analyses of the released fractions, this has revealed insights into proteins associated with active chromatin, and how proteins relocate on the chromatin depending on the cellular context (25, 26, 27) (Fig. 1, *D* and *E*). An orthogonal approach is to expose native nuclei to different concentrations of salts, as TF binding is largely driven by electrostatic interactions (28). MS analyses of nuclei exposed to increasing salt concentrations revealed that euchromatic factors are released at lower salt concentrations (∼250 mM NaCl), whereas heterochromatin proteins require a higher salt concentration (∼600 mM NaCl) (29) (Fig. 1*F*). A third approach to separate euchromatin and heterochromatin, called gradient-seq, uses a crosslinking approach comparable to DEMAC and makes use of ultracentrifugation of crude chromatin fragments in a sucrose gradient. This approach separates molecules depending on their sedimentation rate, which is mainly determined by the size of the molecules (Fig. 1*G*). This is in contrast to a cesium chloride gradient, through which molecules diffuse based on their protein–DNA ratio. The gradient-seq method has been used to study difficult-to-sonicate heterochromatin regions, and MS-based analyses of this fraction revealed good concordance with previously described proteins associated with heterochromatic regions (30). These methods can be used in combination with total chromatin proteomes to identify novel TFs and investigate their dynamic distribution across chromatin.

## Isolation of Newly Synthesized Chromatin Strands for MS

When chromatin is isolated from cells or tissues, the sample contains cells that are in different phases of the cell cycle and thus represents a mixture of “old” and “new” chromatin. An open question in the field remains how exactly new chromatin is formed during replication, how DNA replication is integrated with chromatin dynamics, and which chromatin structures and proteins are transmitted to newly formed chromatin. Several research groups have thus taken up the task to specifically isolate and characterize newly synthesized chromatin strands that are generated during replication. This has resulted in two analogous methods called isolation of Proteins On Nascent DNA and nascent chromatin capture (31, 32) (Fig. 1, *H* and *I*), which rely on incubating cells for a short period (10–20 min) with thymidine analogs that are incorporated into newly synthesized DNA strands. In case of isolation of Proteins On Nascent DNA method, the analog is 5-ethynyl-2′-deoxyuridine, which can be covalently linked to biotin–azide after crosslinking, allowing enrichment of nascent chromatin using streptavidin beads. The Nascent Chromatin Capture protocol relies on integration of biotin–2′-deoxyuridine, 5′-triphosphate, which after cell lysis and chromatin isolation can be used directly as a handle for nascent chromatin isolation using streptavidin-coated beads. These protocols have for example revealed the important role of FAM111A as a replication factor for proliferating cell nuclear antigen loading and consequently progression of the cell cycle (31). These protocols of isolating nascent chromatin have now also been adapted to total chromatin extraction, which can be obtained with a similar workflow though extending the labeling time with thymidine analogs to ∼20 h (33) (Fig. 1*H*). The resulting so-called DNA-mediated chromatin pull-down workflow is easy to perform with limited reagents, provided that the cell type of interest is proliferating.

Altogether, many workflows have been developed to study the chromatin proteome, and the choice of method should be tailored to the research question, the available infrastructure in the laboratory, the amount of biological material available, and whether the study focuses on cultured cells or tissue material (Fig. 1 and Table 1).

**Table 1:** Overview of approaches to isolate total chromatin, nascent chromatin, or euchromatin/heterochromatin for proteomics approaches

Technique	# Cells input	Xlink	Brief description	Experiment type	Applicable to tissues	Live cells required	References
Crude fractionation	10^6^–10^8^	No	Cellular fractionation into cytoplasmic, nuclear and chromatin fractions	Total chromatin	No	Yes	(10, 11, 13)
ChEP	10^7^	Yes	Differential extraction under denaturing condition	Total chromatin	Yes	No	(14)
DEMAC	10^8^	Yes	Bouyant density separation using cesium chloride ultracentrifugation	Total chromatin	Yes^a^	No	(20)
Dm-ChP	10^6^	Yes	EdU labeling (20 h), biotin click, and streptavidin enrichment	Total chromatin	No	Yes	(33)
D-CAP	10^6^	No	Nuclear washes and MNase digestion	Total chromatin (tightly bound)	No	Yes	(26)
Differential MNase	10^6^	No	Differential MNase digestion	Euchromatin/heterochromatin	No	Yes	(25, 27)
CHESS-DIA	10^6^	No	Differential salt extraction	Euchromatin/heterochromatin	No	Yes	(29)
Gradient-seq	10^8^	Yes	Differential ultracentrifugation in sucrose gradient	Euchromatin/heterochromatin	Yes^a^	No	(30)
iPOND	10^8^	Yes	EdU labeling (10 min), biotin click, and streptavidin enrichment	Nascent chromatin	No	Yes	(32)
NCC	10^8^	Yes	biotin-dUTP incorporation and streptavidin enrichment	Nascent chromatin	No	Yes	(31)

dUTP, 2′-deoxyuridine, 5′-triphosphate; EdU, 5-ethynyl-2′-deoxyuridine; iPOND, isolation of Proteins On Nascent DNA; NCC, nascent chromatin capture; CHESS-DIA, chromatin enriching salt separation coupled to data independent acquisition.

“# Cells input” indicates the estimated number of cells that should be used per replicate. “Xlink” indicates whether crosslinking using formaldehyde should be applied. Applicable to tissue indicates whether this method could be used on, for example, tissue sections, organs, or biopsies that are derived from animals or patients.

a Indicates that the techniques could be used on tissues provided nuclear isolation can be performed and sufficient quantities can be obtained. Note that in principle these techniques could be used on cells obtained from any eukaryotic organism.

## Enrichment of Localized Chromatin Compartments

Analysis of the chromatin proteome is highly informative and can be used to identify candidate regulatory chromatin factors for cell types of interest as well as their dynamics. How these TFs act to regulate cell type–specific gene expression can be further investigated using genomics tools such as CHromatin Immuno Precipitation followed by sequencing or cleavage under targets and release using nuclease. This can be combined with TF perturbation followed by gene expression profiling, which yields a global picture as to if, and to some extent how, this TF is relevant for a specific cell type. However, to understand in detail how a TF functions, it is important to understand the chromatin environment in which it is functional, to identify relevant PPIs, to determine how local chromatin features define PPIs, and how this is affected by a (disease-induced) mutation in the protein itself or the DNA it associates with. A wide range of methods are currently available to study protein–protein and protein–DNA binding of chromatin factors. These assays frequently use crude nuclear extracts, followed by either affinity purification (AP) of a TF of interest using antibodies or introduced protein tags such as GFP (8). Alternatively, nuclear extracts can be incubated with histone tails carrying a specific modification (34), reconstituted (un)modified nucleosomes (35), or a DNA sequence harboring a specific DNA motif to identify the TFs that associate with this sequence (36, 37, 38). While these assays are informative to obtain DNA-binding specificity, histone (modification) binding potential, or to identify protein interaction partners, these assays have their limitations. They do not discriminate between interactions occurring on chromatin or in the nucleoplasm, and how this might modulate the stoichiometry of a protein complex. The absence of a surrounding chromatin environment mitigates the effect of TF binding to nearby DNA or histone modifications. In addition, TFs may be crowded through the local 3D chromatin structure or phase separation of chromatin compartments, potentially affecting their behavior and binding partners. This is substantiated by the notion that protein complexes on and off chromatin are vastly different (39). Therefore, in recent years, a range of MS-based methods have been developed to study TFs and TF proximal proteins on chromatin *in vivo*.

Initial efforts to characterize the local proteome of a TF bound to chromatin made use of tandem AP–tagged proteins in budding yeast, by first lysing cells in 300 mM NaCl buffer and subsequently digesting DNA with DNaseI (40). By performing these purifications iteratively, the authors identified functional units within their data set such as the presence of YTA7 at some boundary elements between active and silent chromatin. A variation on this approach was to lyse cells in low salt (100 mM potassium chloride) buffer without DNaseI treatment but to employ sonication to solubilize protein complexes still associated with relatively large DNA fragments (∼1 kb). Using the protein A moiety of the tandem AP tag as an affinity handle, protein interaction networks, containing both direct and indirect (through chromatin) interactions, could be characterized (41). This modified ChIP method (referred to as mChIP) was successfully applied to a large array of yeast chromatin-associated proteins (42). The general principle of preserving the protein–DNA association throughout the biochemical purification of specific complexes prior to MS analyses, with or without crosslinking, has been adapted for use in numerous organisms including *Drosophila* (43) and human cells (44, 45).

**Fig. 2:**
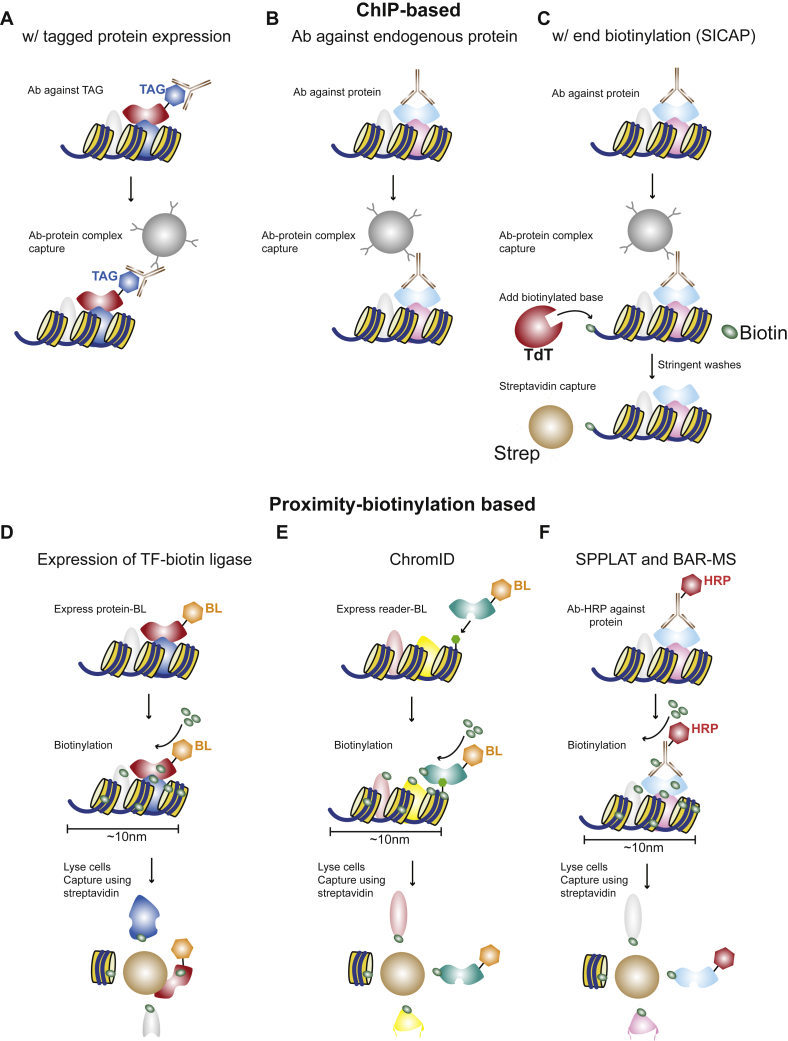
**Strategies for analyzing the proximal proteome of a chromatin protein of interest**. *A*–*C*, ChIP-based approaches using tagged protein expression (*A*, ChIP–MS, mChIP, BioTAP-XL, and native chromatin capture), antibodies against endogenous proteins (*B*, ChIP–MS, RIME, qPLEX-RIME, Q-TIP, ChroP) and the same procedure as in (*B*) but followed by end-biotinylation *via* terminal deoxynucleotidyl transferase (TdT) and capture (*C*, SICAP). *D*–*F*, proximity-biotinylation methods using expression or tagging of a protein with a biotin ligase (*D*), expression of a reader domain for a chromatin mark (in *light green*), fused to a biotin ligase (*E*), or primary (or secondary) antibodies fused to HRP (*F*). BL indicates biotin ligase. Note that the images are illustrative and that the 10 nm indicated for panels *D*–*F* indicates the biotinylation distance, not *per se* that three nucleosomes span 10 nm. It should also be noted that although 10 nm is the labeling distance previously observed for nuclear pore complexes, the exact labeling distance that biotin ligases can achieve on chromatin still has to be experimentally addressed. ChIP–MS, CHromatin Immuno Precipitation–MS; ChroP, chromatin proteomics; HRP, horse radish peroxidase; mChIP, modified ChIP; RIME, Rapid Immunoprecipitation MS of Endogenous proteins; SICAP, selective isolation of chromatin-associated proteins.

These so-called ChIP–MS workflows rely on introducing tagged proteins into cells. However, this may be challenging in cell types that are hard to transfect or already fixed and could introduce artifacts because of (over)expression of the tagged protein. This required adaptations of the ChIP–MS workflow to use antibodies to a protein of interest, resulting in methods referred to as chromatin proteomics (ChroP) (46), quantitative telomeric chromatin isolation protocol (47), ChIP–MS (48, 49), Rapid Immunoprecipitation MS of Endogenous proteins (RIME) (50), and an improved and more scalable version of RIME, quantitative multiplexed RIME (qPLEX-RIME) (51) (Fig. 2, *A* and *B*). These methods use analogous principles as mChIP but only require an antibody for a protein of interest and a cell type that can be obtained in sufficient quantities, making them implementable in virtually every laboratory, and as such, these assays are frequently used nowadays. While antibody-based methods may also be subject to biases caused by nonspecific binding of antibodies or blocking of the epitope recognized by the antibody, they have the advantage of being able to target post-translational modifications on histones or other proteins.

However, these ChIP-based approaches also have some limitations. At first, application of these methods does not involve a chromatin isolation step but rather uses sonicated nuclei as input for the IP. This results in a higher degree of contamination from “hitchhiker” proteins binding to the highly charged DNA backbone (18) and allows purification of antibodies associated with proteins that are not bound to chromatin, which might result in higher abundance of known contaminant proteins such as proteins binding to RNA and ribosomal proteins through crosslinking artifacts (52). In addition, these methods use a relatively large amount of antibodies (generally 2–5 μg), which are also measured during MS analysis and which may suppress peptide signals from chromatin proteins. In principle, contaminant proteins do not pose a major problem, provided the bait is specifically enriched and sequenced along with its interaction partners, and proper outlier statistics can be performed to identify enriched proteins. However, antibody-derived peptides do interfere with the MS measurement, thereby masking low-abundant and small, more difficult to quantify, proteins. Furthermore, it should be noted that the use of antibodies in nonfixed material targeting an epitope within a protein surface mediating PPIs may also compete with interaction partners for binding, resulting in incomplete protein interaction networks being observed.

To overcome these issues, the ChIP and selective isolation of chromatin-associated proteins (ChIP–SICAP) method was developed (53). This approach builds on the ChIP–MS workflow, but after the IP step, the obtained DNA fragments are labeled with biotin using a terminal deoxynucleotidyl transferase using biotinylated nucleotides. This allows enriching protein–DNA fragments on streptavidin-coated beads, while washes with a high concentration of detergents allow removal of antibodies and other remaining contaminants (Fig. 2*C*). ChIP–SICAP has been successfully used to identify proteins associated with the pluripotency network in embryonic stem cells, revealing TRIM24 as a novel pluripotency-associated protein (53). While the ChIP–SICAP procedure is somewhat more laborious than ChIP–MS, it yields fewer contaminant proteins while simultaneously obtaining higher intensities for expected proteins in direct comparisons to ChIP–MS and RIME and thus currently seems to yield the “cleanest” MS data (53) (Table 2).

**Table 2:** Overview of methods used for enrichment of local chromatin proteomes

Technique	Organism used	# Cells input	Brief description	Xlink	Transf. required	Appl. to PTMs	Reference
Native chromatin capture	Yeast	10^7^	Endogenous protein tagging or expression of tagged chromatin-domain specific proteins	No	Yes	No	(45)
mChIP	Yeast	10^10^	Endogenous protein tagging or overexpression of tagged chromatin proteins of interest	Yes/no	Yes	No	(41)
ChIP–MS	*Drosophila*	10^9^	Introduction of MSL–HTB fusion proteins	Yes	Yes	No	(43)
BioTAP-XL	Mammalian/*Drosophila*	10^8^–10^9^	Expression of BioTAP-tagged proteins of interest	Yes	Yes	No	(44)
ChroP	Mammalian	10^8^	Antibody to protein of interest	Yes/no	No	Yes	(46)
Q-TIP	Mammalian	10^7^–10^8^	Antibody to protein of interest	Yes	No	Yes	(47)
ChIP–MS	Mammalian	10^8^	Antibody to protein of interest	Yes	No	Yes	(48, 49)
RIME	Mammalian	10^7^	Antibody to protein of interest	Yes	No	Yes	(50)
qPLEX-RIME	Mammalian	10^6^	Antibody to protein of interest	Yes	No	Yes	(51)
SICAP	Mammalian	10^7^	Antibody to protein of interest, DNA-end biotinylation, and streptavidin enrichment	Yes	No	Yes	(53)
Proximity biotinylation	Mammalian/plant/yeast	10^6^–10^8^	Tagging of protein of interest with a biotinylating protein fragment (BioID/APEX2/TurboID)	No	Yes	No	(58, 59, 60)
ChromID	Mammalian	10^7^	Expression of chromatin-reader domains coupled to a biotin ligase	No	Yes	Yes	(64)
BAR-MS and SPPLAT	Mammalian	10^6^	Primary antibody, secondary HRP conjugated, biotinylation, and streptavidin enrichment	Yes	No	Yes	(65, 66)

BAR-MS, biotinylation by antibody recognition; SPPLAT, selective proteomic proximity labeling assay using tyramide.

“# Cells input” indicates the number of cells that have to be used per replicate. “Xlink” indicates whether crosslinking with formaldehyde should be used. “Transf. required” indicates whether cells have to be transfected. “Appl. to PTMs” indicates whether the method can be applied specifically to post-translational modifications.

## The Proximity Proteome of Chromatin Proteins Using Proximity Biotinylation

Next to potential crosslinking artifacts induced in ChIP-based methods, these also require sonication of chromatin. This is a variable process, and it is not trivial to reproducibly obtain fragments of the same length, which in turn can lead to variations between experiments in terms of ChIP efficiency and identified proteins (54). In addition, several of the ChIP-based protocols are quite elaborate and take several days to perform. An attractive alternative to ChIP-based approaches could be the use of proximity biotinylation. This methodology relies on fusing a biotin ligase to a protein of interest, which allows to label proteins in a 10 nm range with biotin (55, 56). Using proximity biotinylation has some major advantages in terms of sample handing, as after the biotinylation reaction, cells can be lysed and the extract can be directly incubated with streptavidin-coated beads (Fig. 2*D*). Furthermore, since bait-proximal proteins become biotinylated, denaturing conditions can be used during cell lysis and subsequent affinity enrichment. As the biotin–streptavidin interaction is extremely strong, the IP and the washes can therefore be performed in the presence of high concentrations of salt and detergents, which strongly reduce the number of contaminants. The most commonly used enzymes are an engineered soybean ascorbate peroxidase called APEX2, and a promiscuous mutant of the *Escherichia coli* biotin ligase BirA, referred to as BioID, which has recently been derived to an enhanced and much faster enzyme called (mini)TurboID (extensively reviewed in (57)). Of these, APEX2 and (mini)TurboID require the shortest labeling time (1–10 min) and are therefore currently the enzymes of choice for time-resolved proximity labeling workflows. It is worth nothing that the use of a slower biotin ligase enzyme may enable the biotinylation reaction to occur across multiple cellular contexts (*e.g.*, cell cycle stages) enabling the identification of partners that may potentially be missed by using a faster enzyme. While such assays can be used for any protein of interest, they are particularly useful to investigate the local proteome of chromatin-bound proteins (58, 59, 60). Such biotinylation assays can be further tailored to address different questions, for example, to determine the local proteome that is associated with two chromatin factors of interest (*e.g.*, a TF and a chromatin modifier) when these are in close proximity *in vivo*. In such cases, the biotin ligase can be “split” over the two chromatin proteins, and only when these are close together, the enzymatic activity is reconstituted, which then results in biotinylation of the proximal environment (61, 62, 63). This principle has been used to obtain the proximity proteome of the contact site between the endoplasmic reticulum and the mitochondria (63). A recent study further highlighted the value of proximity labeling by fusing biotin ligases to protein reader domains that can recognize chromatin modifications, which provides a promising tool to identify proteins in the proximity of nontaggable protein forms such as a histone mark (64) (ChromID; Fig. 2*E*). Finally, proximity biotinylation can also be performed by horseradish peroxidase (HRP), which is an enzyme that can be coupled to antibodies. By targeting an antibody–HRP conjugate to proteins inside cells, a proximal proteome for any protein of interest can be obtained, provided a specific antibody is available (65, 66) (selective proteomic proximity labeling assay using tyramide and biotinylation by antibody recognition; Fig. 2*F*). This approach has already been shown to be applicable to chromatin factors (66), though it should be noted that this approach only works in fixed material as HRP is not as functional in the cytosol or other reducing environments of the cell (67).

Taken together, a large toolbox is now available to investigate local chromatin environments for any given protein of interest, and the choice of method depends on the amount of available material, the organism used, whether the cell type of interest can be genetically modified, and if suitable antibodies are available. In addition, only those methods involving an antibody or ChromID, given the reader domain and target are specific, can be directed to specific post-translational modifications (Table 2 and Fig. 2). In general, while some comparisons have been made between ChIP-based methods, it will be critical to evaluate the performance of these workflows compared with biotinylation-based approaches. Important aspects to assess will be fold enrichment of the bait and associated factors over control samples and the degree of contamination from nonchromatin proteins.

## The Biochemical Challenges and Opportunities

The discussed methods for ChroP require a relatively large amount of input material, ranging from several millions to hundreds of millions of cells (Tables 1 and 2). However, a great challenge for ChroP, or proteomics studies in general, is that many biological samples, such as embryos, organoids, and clinical material, can only be retrieved in limited quantities. As such, adaptations of the workflows are required to facilitate applications with low-input samples. One recent innovation toward this aim is a microfluidics-based AP–MS platform, which can be used to identify PPIs from as little as 12.000 input cells (68). Additional workflow adaptations related to sample preparation prior to MS-based analysis may also be considered. One of the potential approaches could be to integrate a single-pot solid-phase enhanced sample preparation procedure in chromatin proteomics applications (69). This method uses paramagnetic beads to efficiently capture proteins and peptides in a single tube, and this workflow has already been incorporated in the previously described ChIP–SICAP procedure (53). Finally, at the level of the mass spectrometer, a range of new data acquisition methods are available that should be particularly suitable for low-input applications (70). An important avenue to explore is data-independent acquisition (DIA). For a long time, data-dependent acquisition, which relies on automated instrument control for MS/MS acquisition, has been the standard acquisition method for MS. In data-dependent acquisition, peptides are sequenced based on peptide abundance, which means that very low–abundant peptides are often either not sequenced or masked by high-abundant peptides. This might especially pose a problem in MS-based analyses of chromatin because of the presence of high-abundant histone proteins. With DIA, peptides within a specified *m*/*z* range are fragmented comprehensively, irrespective of the abundance of the peptides in that *m*/*z* range (71). Thus, in principle, DIA should offer a more comprehensive analysis of peptides and as a consequence could be more suitable for MS analysis of chromatin (domains). Finally, when specific chromatin factors or TFs are subject of study, selective reaction monitoring can be used to specifically measure the abundance of tens to hundreds of preselected proteins over a wide range of abundances (72).

Further research is required to optimize and implement these MS data acquisition methods for low-input chromatin proteomics studies, but important lessons can perhaps be learned from the emerging field of single-cell proteomics, which deals with extremely low amount of input sample (73).

A second challenge lies in the scalability of chromatin proteomics workflows. While high-throughput workflows for deep proteome analyses are available (74), development of high-throughput applications for more targeted approaches such as chromatin(-domain) proteomics is still lagging behind. Such workflows may be highly desirable to decipher the dynamic composition of a given chromatin domain upon cellular stimulations or perturbations. A notable example comprises the telomeres. These structures protect the ends of chromosomes and are essential for maintenance of cellular integrity. Furthermore, telomere maintenance is frequently affected in diseases such as cancer. Targeting of telomeres and the telomere-lengthening enzyme, telomerase reverse transcriptase, provides potential therapeutic avenues in the treatment of cancer (75). To understand how telomere-targeting drugs function, it is relevant to investigate how, and if, they affect binding of important telomere-binding proteins such as the shelterin complex. Telomeres (and other repetitive elements) can be efficiently purified for MS analyses using complementary biotinylated locked nucleic acid probes, although this currently requires relatively large amounts of input material (10^8^–10^9^ cells) (76, 77), thus compromising automation and high-throughput applications. For ChIP-based methods, automated workflows are available (78, 79), but it is not clear whether these will allow purifying sufficient protein amounts for MS-based studies. In this respect, proximity biotinylation might provide a good alternative, given the relatively simple workflow, omission of crosslinking, and possibility for stringent washes. As such, it is conceivable that coupling streptavidin IPs to automated microfluidics systems will allow high-throughput chromatin domain proteomics upon perturbations such as drugs targeting epigenetic enzymes. In addition, the use of isobaric labeling approaches such as tandem mass tags will allow sample multiplexing without compromising peptide detection, thus allowing increased throughput without increasing MS measurement time.

Finally, a much-discussed challenge in the chromatin field is single-locus proteomics. The biology of a single locus in the genome such as a promoter or an enhancer can only be completely understood if the proteome of that locus is known. While approaches are available that target repetitive elements such as telomeres in a reproducible manner (76, 77), and advances are being made toward purifying a single genomic locus, there are still substantial improvements required to accurately determine the local proteome of a nonrepetitive locus (reviewed in (70, 80)). A possible intermediate solution between ChIP–MS and single-locus proteomics could be the use of the previously mentioned split biotin ligase enzyme. In this scenario, one part of the enzyme may be fused to a TF of interest and the other end to a locus-specific protein or process-specific protein. As an example, the epigenetic protein complex nucleosome remodeling and deacetylase (NuRD) localizes to many loci to not only regulate gene expression but also gets recruited to sites of DNA damage (81). A split biotin ligase approach could be to fuse the enzyme parts to a NuRD subunit and a DNA damage protein, which would allow determining the NuRD proximal proteome in the context of the DNA damage response. An orthogonal approach could be to label specific proteins such as histone variants with a biotin acceptor peptide and fuse a TF with the BirA ligase, which only biotinylates this acceptor peptide, which works well in sequencing-based experiments (82, 83). Coexpression of these two proteins would then allow a ChIP–MS like experiment but using the biotinylated acceptor peptide as affinity enrichment handle. This would then result in enrichment and analysis of the chromatin proteome at regions where these two proteins are in close proximity.

## Concluding Remarks and Future Perspectives

Recent developments in the chromatin proteomics field have allowed analysis of the entire chromatin proteome or subfractions thereof. These approaches, also when integrated with genomics approaches, will form a strong basis to discover novel TFs associated with cellular homeostasis, development, and disease.

Epigenetic proteins and complexes are frequently studied by determining their composition and architecture in crude cell extracts or in solution. However, the behavior of proteins is often different as soon as they are taken out of their native contexts, underlined by the existence of distinct versions of protein complexes on and off chromatin (39). In the future, it will thus be important to assess to what extent PPIs obtained using AP–MS approaches also occur when proteins are associated with chromatin. This also applies to proximity biotinylation assays. In standard protocols, cells are lysed in a denaturing buffer after the biotinylation reaction, after which whole cell lysates are incubated with streptavidin beads. A thorough comparison of bait-proximal biotinylation in the nucleoplasm *versus* the chromatin fraction is thus an important goal for the future.

It is further likely that the 3D organization of protein complexes changes when they become associated with a chromatin template. It would therefore be highly informative to determine the topology and the 3D structure of proteins and protein complexes bound to chromatin. One approach would be to combine crosslinking–MS (XL–MS) with any of the chromatin-domain enrichment procedures described previously. While potentially biochemically challenging, XL–MS has readily been shown to be applicable to intact nuclei (84), providing an important step toward obtaining crosslinking maps of enriched chromatin fragments. If XL–MS could be adapted for application in chromatin enrichment workflows, this will have an extra benefit as it allows to distinguish direct from indirect protein interactions, which is currently not yet possible with the described chromatin (domain) enrichment strategies. This could be complemented with cryoelectron microscopy maps of protein complexes reconstituted from purified proteins or directly obtained from the native (chromatin) environment (85, 86).

Another important aspect of chromatin biology that is becoming increasingly apparent is phase separation. The process of phase separation yields liquid condensates that can confine protein and nucleic acids in separate membraneless compartments. Recent advances have demonstrated that reconstituted chromatin fibers can phase separate and that this can be modulated by histone modifications (87). In addition, several TFs have been shown to undergo phase separation, such as the integral heterochromatin protein 1 (88) and the mediator–complex subunit 1, MED1 (89). As aberrant phase separation is observed in diseases such as ALS and can arise from protein fusions with TFs in cancer, it will be relevant to assess how phase separation of a TF affects the (local) chromatin proteome. As membraneless organelles are sensitive to detergents, especially proximity biotinylation assays for chromatin factors in phase separation promoting and disturbing conditions will provide a powerful tool.

While this review mainly focuses on TFs, the epigenome is highly complex. Many different RNA molecules are associated with the chromatin, and the DNA, as well as RNA and proteins associated with it, can be chemically modified. In terms of chromatin extraction, informative assays will be to use the techniques described in this review to assess whether proteins and RNA possess specific modifications when bound to chromatin (compartments). This information will be required to advance our understanding of chromatin regulation at the local scale, and how this is for example dynamic in cell fate transitions or perturbations.

In summary, because of improvements in hardware, software, and methodology, we envision that chromatin proteomics will take up a place in routine epigenetic research. Future improvements aimed at downscaling will allow obtaining global and local chromatin proteomes, even when small amounts of input material are available. Finally, efforts aimed at increasing throughput will allow screens to investigate how drugs or compounds modulate the (local) chromatin proteome.

## Conflict of interest

The authors declare no competing interests.
